# The attribution of human health outcomes to climate change: a transdisciplinary guidance document

**DOI:** 10.1007/s10584-025-03976-7

**Published:** 2025-07-23

**Authors:** K. Ebi, A. Haines, R. F.S. Andrade, C. Åström, M. L. Barreto, A. Bonell, N. Brink, C. Caminade, C. J. Carlson, R. Carter, P. Chua, G. Cissé, F. J. Colón-González, S. Dasgupta, L. A. Galvao, M. Garrido Zornoza, A. Gasparrini, G. Gordon-Strachan, S. Hajat, S. Harper, L. J. Harrington, M. Hashizume, J. Hess, J. Hilly, V. Ingole, L. V. Jacobson, T. Kapwata, C. Keeler, S. A. Kidd, E. W. Kimani-Murage, R. K. Kolli, S. Kovats, S. Li, R. Lowe, D. Mitchell, K. Murray, M. New, O. E. Ogunniyi, S. E. Perkins-Kirkpatrick, J. Pescarini, B. L. Pineda Restrepo, S. T. R. Pinho, V. Prescott, N. Redvers, S. Ryan, B. Santer, C.-F. Schleussner, J. C. Semenza, M. Taylor, L. Temple, S. Thiam, W. Thiery, A. M. Tompkins, S. Undorf, A. M. Vicedo-Cabrera, K. Wan, R. Warren, C. Webster, A. Woodward, C. Wright, R. F. Stuart-Smith

**Affiliations:** 1 University of Washington Center for Health and the Global Environment; 2 London School of Hygiene and Tropical Medicine; 3 Center for Data and Knowledge Integration for Heath-Cidacs, FIOCRUZ; 4 Umeå University; 5 Center for Data and Knowledge Integration for Heath-Cidacs, FIOCRUZ; 6 London School of Hygiene & Tropical Medicine; 7 Wits Planetary Health Research, University of the Witwatersrand, Johannesburg, South Africa, and Department of Public Health and Primary Care, Trinity College Dublin, Dublin, Ireland; 8 The Abdus Salam International Centre for Theoretical Physics; 9 Yale University; 10 University of Washington; 11 The University of Tokyo; 12 (1) Centre Suisse de Recherches Scientifiques en Côte d’Ivoire (CSRS), (2) Swiss Tropical and Public Health Institute (Swiss TPH), (39 University of Basel (Unibasel); 13 Wellcome Trust; 14 Fondazione CMCC; 15 Oswaldo Cruz Foundation - Fiocruz/CRIS; 16 International Centre for Theoretical Physics (ICTP); 17 London School of Hygiene & Tropical Medicine; 18 University of the West Indies; 19 London School of Hygiene & Tropical Medicine; 20 University of Alberta; 21 Te Aka Mātuatua School of Science, University of Waikato, New Zealand; 22 The University of Tokyo; 23 University of Washington; 24 School of Civil and Environmental Engineering, University of New South Wales; 25 Office For National Statistics; 26 Fluminense Federal University; 27 South African Medical Research Council; 28 University of Washington; 29 Centre for Addiction and Mental Health/ University of Toronto; 30 African Population and Health Research Center (APHRC); 31 International Monsoons Project Office; 32 London School of Hygiene & Tropical Medicine; 33 The University of Sheffield; 34 Barcelona Supercomputing Center (BSC-ICREA); 35 University of Bristol; 36 MRC Unit The Gambia @ LSHTM; 37 University of Cape Town; 38 Climate Risk Laboratory, University of Cape Town; 39 Fenner School of Environment and Society, Australian National University; 40 London School of Hygiene & Tropical Medicine; Oswaldo Cruz Foundation; 41 Organismo Andino de Salud-Convenio Hipólito Unanue; 42 Federal University of Bahia; 43 Australian Intitute of Health and Welfare; 44 Schulich School of Medicine & Dentistry, University of Western Ontario; 45 University of Florida; 46 Woods Hole Marine Biological Laboratory; 47 International Institute for Applied System Analysis (IIASA); 48 Umeå University; 49 University of the West Indies; 50 Oxford Sustainable Law Programme, University of Oxford; 51 African Population and Health Research Center, West Africa Regional Office; 52 Vrije Universiteit Brussel; 53 International Centre for Theoretical Physics (ICTP); 54 Potsdam Institute for Climate Impact Research (PIK); 55 Institute of Social and Preventive Medicine, University of Bern, Bern, Switzerland // Oeschger Center for Climate Change Research, University of Bern, Bern, Switzerland; 56 London School of Hygiene & Tropical Medicine; 57 Tyndall Centre for Climate Change Research, University of East Anglia, Norwich, UK; 58 South African Medical Research Council and University of Pretoria; 59 University of Auckland; 60 South African Medical Research Council; 61 Oxford Sustainable Law Programme, University of Oxford, UK

## Abstract

For over 30 years, detection and attribution (D&A) studies have informed key conclusions in international and national assessments of climate science, providing compelling evidence for the reality and seriousness of the human effects on the global climate. In the early 21st century, D&A methods were adapted to assess the contribution of climate change to longer-term trends in earth system processes and extreme weather events. More recently, attribution research helped quantify the health and economic impacts of climate change. Here we provide guidance for transdisciplinary collaboration in designing, conducting, interpreting, and reporting robust and policy-relevant attribution analyses of human health outcomes. This guidance resulted from discussions among experts in health and climate science. Recommended steps include co-developing the research question across disciplines; establishing a transdisciplinary analytic team with fundamental grounding in the core disciplines; engaging meaningfully with relevant stakeholders and decision-makers to define an appropriate study design and analytic process, including defining the exposure event or trend; identifying, visualizing, and describing linkages in the causal pathway from exposure to weather/climate variables to the health outcome(s) of interest; choosing appropriate counterfactual climate data, and where applicable, to evaluate the skill of the climate and process or empirical health model(s) used in D&A research; quantifying the attributable changes in climate variables; quantifying the attributable health impacts within the context of other determinants of exposure and vulnerability; and reporting key results, including a description of how recommendations were incorporated into the analytical plan. Implementation of guidance would benefit diverse stakeholders including researchers, research funders, policymakers, and climate litigation by harmonizing methods and increasing confidence in findings.

## 1 Introduction

Detection and attribution (D&A) analyses underpin the conclusions since the mid-1990s that human activities have unequivocally warmed the atmosphere, ocean, and land (IPCC, 2021). Trend attribution methods quantify the proportion of an observed change in the Earth system from a pre-industrial baseline period that may be causally attributed to specific anthropogenic forcings (e.g., well-mixed greenhouse gases and aerosols; Santer et al., 2003; Hegerl et al., 2010; Stone et al., 2013, Eyring et al., 2021). Climate scientists have applied these methods to quantify the extent to which greenhouse gas emissions have caused changes in surface temperature (e.g., Gillett et al., 2021), different components of the hydrologic cycle, glacier retreat, sea-level rise, and many other variables (see Eyring et al., 2021, and references therein). For extreme weather and climate events, related methods commonly assess the change in an event’s intensity and/or frequency attributable to anthropogenic climate change (Stott et al., 2004; Seneviratne et al., 2021).

Recent developments extended the application of attribution methods to assess impacts on different sectors (O’Neill et al., 2022). Examples include economic losses, food security, wildfires, agriculture, hydrological change, and health (e.g., Uhe et al., 2021, Mitchell et al., 2016, Ebi et al., 2017; Ebi et al., 2020; Dasgupta and Robinson, 2022; Romanovska et al., 2024; Burton et al., 2024; Gibb et al., 2023; Vicedo-Cabrera et al., 2021, 2023; Stuart-Smith et al., 2024). However, existing health attribution studies are still limited, both in terms of the health impacts covered and their geographical scope (Carlson et al., 2024). Current understanding of the health impacts of climate change is consequently partial, biased towards types of exposures where the climate science community has conducted D&A analyses and towards regions with more abundant long-term health and weather data. D&A analyses are starting to focus on places assumed to be the most vulnerable and most affected by climate change (e.g. Carlson et al., 2024; Callaghan et al., 2021; Childs et al., 2024). In practice, most health attribution studies have concentrated on problems where the anthropogenic signal dominates the noise in the climate system, and where the health outcome is strongly associated with climate variables (e.g. heat-mortality).

The definition of attribution can vary between and within the climate science and impact communities. Climate science typically focuses on attribution of changing weather/climate patterns to radiative forcings and sometimes to specific emitters of greenhouse gases. Impact communities focus on attribution to radiative forcings or on other metrics of climate change (e.g., extreme weather and climate events). Health attribution studies have focused on the latter.

Incomplete understanding and documentation of the impacts of climate change on health impedes decision-making and legal responses. D&A studies could be an important input for climate risk assessments to inform policy; and could provide leverage for implementing adaptation policies for health, agriculture, and other sectors, to support affected individuals and communities. Improved and expanded evidence on health impact attribution might also inform litigation and the program of the nascent Loss and Damage Fund established by the United Nations Framework Convention on Climate Change (UNFCCC) at COP27 (UNFCCC 2022).

To increase the robustness and relevance of D&A studies of human health outcomes, we developed a framework to promote robust D&A study design and analyses that follow best practices, based on evidence and expert knowledge. The framework is designed to promote transparency on analytic choices made, not to provide a rigid approach. Within this framework, we include guidance for designing, conducting, interpreting, and reporting D&A analyses. This guidance is intended to support transdisciplinary collaborations to improve methods, including new approaches where necessary, and to build the capacity for conducting empirical studies, including in low-resource settings, that, in turn, contribute to national and international assessments of the impacts of climate change on human health. In addition to researchers in health, climate, and other disciplines, the intended audiences of this framework include editors of academic journals, research funding organizations, public health organizations, experts involved in ongoing scientific assessments, decision-makers, and legal scholars. The guidance is designed so that future iterations can easily incorporate diverse knowledge systems and new scientific developments.

## 2 Methods

Three steps were taken to develop and refine the framework and associated guidance for conducting robust and policy-relevant health D&A analyses:

The first step involved recruitment of an international panel of scientific experts. The organizing team (K.L. Ebi, R.F. Stuart-Smith, A. Haines, J.J. Hess) identified a scientific advisory committee based on expertise across issues in health and climate attribution (C. Wright, L.A. Galvao, M. Taylor, and R.K. Kolli). The organizing and scientific advisory committees selected 65 scientists from a systematic review of the scientific literature and snowball sampling to potentially participate in a hybrid workshop intended to generate consensus recommendations on a framework and guidance on D&A analyses for climate change health impacts. Selection criteria included area of expertise (health and/or climate), geographic area of research, gender, and career stage. Facility constraints meant the invitation list could not include all relevant researchers. Expert solicitation, including workshop availability, was conducted by email in August 2024 and substitutions made via mutual agreement between the organizing team and the contacted expert.

In the second step, panelists convened remotely or in person in London, U.K. in a workshop in September 2024 sponsored by the Wellcome Trust and co-convened with the University of Washington. Of the final panelist pool, 35 participated in person and 20 participated remotely. All consenting participants are included as authors. The overarching goal was to reach consensus on key recommendations for a framework and guidelines on conducting robust and policy-relevant climate and health D&A studies. Panel discussions included topics such as making D&A relevant across diverse communities, incorporating novel data sources, prioritizing different types of (qualitative and quantitative) data, loss and damage, and methodological issues. Small group discussions included climate science and public health perspectives on D&A guidance, a narrow focus on climate change vs. planetary health, approaches to stakeholder engagement, and when and how attribution evidence might be used. The agenda and list of participants are available upon request.

In the third step, workshop volunteers formed a core writing team to draft the guidance, which was then circulated and agreed upon by all authors.

## 3 Overview of pathways by which climate change affects health and well-being

Changing weather patterns because of climate change can affect human health and well-being through a wide range of pathways. These include the effects of extreme weather and climate events such as heatwaves, floods, droughts, and wildfires. Long-term changes in weather variables, sometimes combined with extreme events, are altering the prevalence and distribution of water and nutrition-related health outcomes; changes in transmission and emergence of vector-borne, water-borne, food-borne and zoonotic diseases; exacerbation of chronic diseases (e.g. metabolic and allergic); and the effects on mental and physical health from population displacement and poverty (Cisse et al., 2022). Illustrative examples of the plethora of possible causal chains between weather/climate exposures and associated health outcomes.

● Climate hazards associated with extreme weather and climate events such as extreme heat, floods, droughts, typhoons, and wildfire can cause excess morbidity, mortality, mental ill-health and other adverse health effects
● Exposure to higher ambient temperatures can result in morbidity and mortality, including adverse maternal, neonatal and child health outcomes
● Exposure to reduced air quality (from increased air pollution, including due to wildfires, and aeroallergens) can cause a range of health outcomes, including allergic and other respiratory diseases
● Changes in temperature, precipitation, and other weather variables can alter the spatial distribution, seasonality, and/or incidence of a range of infectious diseases, vectors, and disease organisms
● Exposure to climate-driven changes in water and food safety, availability, and security can affect associated disease outcomes

Briefly, these exposures can interact with the social determinants of health to alter underlying vulnerabilities and resilience. Climate change-associated reductions in access to and delivery of healthcare can compound observed impacts. Indigenous Peoples and historically minoritized communities, including displaced peoples, suffer disproportionate impacts because of systematic discrimination and injustice leading to an amplification of poor health outcomes.

Health studies have focused more on detection of climate health associations than on attribution. Many effect estimates have been quantified using linear functions and often have been assumed to be uniform in different contexts for health impact assessments, such as the Lancet Countdown (Romanello et al., 2024); both potentially problematic assumptions. Health outcomes are influenced by many factors, of which climate change is only one. It is important to consider other factors, such as changing socioeconomic and demographic circumstances, changes in upstream drivers of health outcomes, and other earth system changes that could be responsible partly or wholly for observed changes in health outcomes. Different types of D&A analyses may require different approaches. It is important that health D&A studies clearly communicate to what the impacts are being attributed.

Modeling should consider nonlinearities between a weather driver and health outcome (Mitchell, 2021). For example, statistically linking flood-related mortality with precipitation indices given known river flood characteristics, or nutrition-related health outcomes with heat indices where a strong pathway through agricultural production is expected, will omit nonlinearities in the response of these interim impacts and the health outcomes. Modelling these interim impacts (river flood, agricultural production, etc.) as exposures for health analyses can integrate additional process understanding and knowledge of their exposure and vulnerability characteristics, including human management, and thereby strengthen the attribution finding (Cotterill et al., 2024)

Ideally, attribution studies should provide evidence of a causal association at each step of the causal chain (Grose et al., 2024). Attribution studies should seek to assess the influence of the range of drivers that contributed to the health outcome(s) of interest or to the geographic range and seasonality of vectors (Harrington et al., 2022). Relevant considerations include whether the status of the health system affected the magnitude and pattern of health outcomes, and the underlying vulnerabilities or protections that could have modified the extent of the impact (Stone et al., 2021; Jézéquel et al., 2024). Nevertheless, studies that only quantify the effect of climate change influence on health outcomes, ‘all else being equal’, are legitimate modes of inquiry, and remain informative in some settings, such as in climate lawsuits where the relevant question might be how the impacts would have differed absence the actions of defendants (greenhouse gas emissions) (Stuart-Smith et al., 2024).

With many steps in causal pathways between climate and health, studies may span the full, or parts of, these pathways. For vector-borne diseases, for example, the outcome analyzed could be the abundance and/or seasonal activity of the vector or disease organisms, disease incidence, and/or associated morbidity or mortality. Only assessing associations between known hazards for health and climate change (e.g., climate change impacts on temperature and precipitation that alter the frequency, intensity, and extent of wildfires) provides partial evidence in support of attributable impacts. Where possible, it would be preferable if analyses extended to include health outcomes (e.g., mortality from wildfire-related particulate matter (Chen et al. 2021).

## 4 Probabilistic and storyline approaches for event and trend attribution

Two broad approaches have been used in D&A studies: storyline and probabilistic (risk-based) estimates. In some cases, these approaches may be closely related; storylines can be considered a special case of some of the probabilistic analysis when, for instance, the initial condition uncertainty is fully constrained (Leach et al., 2024). Several protocols describe these methods (e.g., Philip et al., 2020 for probabilistic methods).

● The storyline approach usually disaggregates the causes of a given event or trend into multiple causal processes (typically, an approximation of the thermodynamic and dynamic components) and then assesses the contribution of anthropogenic climate change to one or more of these processes (see Perkins-Kirkpatrick et al., 2024 for a more detailed overview). A common approach for applying these methods is to condition the analysis on given dynamical conditions (such as a specific atmospheric pressure pattern) and then assess the effects of anthropogenically forced changes to specific variables such as ocean temperatures or atmospheric CO_2_ concentrations in the build-up to the event (e.g., Ermis et al., 2024) or to quantify the changing likelihood of those dynamical conditions occurring (Faranda et al., 2023). Such an approach often (but not always) neglects changes in dynamical processes due to climate change, which can be useful for simplifying the problem, but dynamical processes can be substantially affected by anthropogenic warming and affect health outcomes.
● The probabilistic approach estimates the change in frequency or intensity of an event, with and without anthropogenic climate change. These studies usually assess either or both the change in likelihood of meteorological conditions exceeding a certain value, or the change in intensity of an event of a given probability. Assessing changes in the intensity of an event with a fixed probability of occurrence could also be viewed as a storyline approach.

Often, an observed outcome, the ‘factual’ range of outcomes produced by historical climate model simulations including all human and natural forcings, is compared with the ‘counterfactual’ range of outcomes that might have occurred due to natural forcings alone over the same period. To generate this counterfactual, studies may use historical-natural (‘hist-nat’) simulations, with the most common being from the Detection and Attribution Model Intercomparison Project (DAMIP) (Gillett et al., 2016). Studies also have used statistical regression-based counterfactuals of observations and re-analyses, for instance available through the Inter-Sectoral Impact Model Intercomparison Project (ISIMIP; Mengel et al., 2020) or generated via time series of anthropogenically-driven global temperature change, such as the Global Warming Index (Haustein et al., 2017) (see Table 1). Toolkits such as the KNMI Climate Explorer have been developed to support these analyses (https://climexp.knmi.nl/). The assessed contribution of climate change varies between events; studies have found substantial influence of climate change on heatwaves supported by a strong theoretical understanding of the processes by which climate change affects extreme heat (Domeisen et al., 2022). By contrast, for example, there is less certainty surrounding the attribution of severe convective storms to climate change because of limitations to the accuracy of precipitation in models (Perkins-Kirkpatrick et al., 2024).

Because the associations between weather or climate variables and impacts are often non-linear, it cannot generally be assumed that the proportion of climate-change attributable health impacts is equal to the proportion of a meteorological event’s likelihood that is attributable to human influence (Perkins-Kirkpatrick et al., 2022). Non-linearity also arises from other drivers of the health outcome of interest, from implemented adaptation options (e.g., heatwave early warning systems) and from compounding and cascading risks. For example, a loss of housing or income source can introduce a cascade of impacts through negative feedback loops.

Care needs to be taken when impacts result from the combined effect of multiple climatic variables such as temperature and humidity, including their wider consequences, particularly when undertaking bias correction. Multivariate indicators such as wet-bulb globe or apparent temperature are widely applied (see e.g. Mitchell 2016; use of Fire Weather Index: Kirchmeier-Young et al., 2017). Copula-based statistical approaches are sometimes used to assess the combined effect of multiple meteorological variables (Chiang et al., 2021; Zachariah et al., 2023). These approaches provide a flexible representation of the multivariate distribution without assuming a linear relationship between changes in each of the variables, such as in hydroclimatic analysis that combine temperature and precipitation (e.g., Tootoonchi et al., 2022). Ultimately, the choice of the variables used should be determined by knowledge of the etiology of the health outcome, particularly the contribution of weather/climate variables to the observed impacts, and pragmatic requirements of the impact modelling approach that might determine whether a multivariate indicator or time series of individual variables is required.

Consideration should be given to the climate forcings that are the focus of the analysis (Skeie et al., 2017), including whether the effects of local land cover change should be accounted for, and which anthropogenic greenhouse gas and aerosol emissions to include. For instance, in some regions local or remote aerosol emissions or land-use changes confound some of the impacts expected from greenhouse gas emissions (e.g. Thiery et al., 2020). Similarly, some observed recent warming due to greenhouse gas emissions resulted from reductions in sulphate aerosols that until then had temporarily masked the warming (NASA 2023).

Approaches that quantify the portion of the probability of a certain event occurring that is attributable to climate change (the ‘Fraction of Attributable Risk’ or FAR) and then apply this finding to quantify the portion of attributable health impacts do not account for any changes in the probability of a health impact occurring as the climate hazard changes in magnitude. Further, this approach is extremely sensitive to the spatial and temporal scales chosen (Cattiaux & Ribes, 2018; Leach et al., 2018), which makes simplistic linkages problematic. This results in the entire health impact being represented as a binary phenomenon that either occurs or does not occur. However, the magnitude of many health impacts increases progressively with the magnitude of the climate hazard. For instance, higher temperatures are normally associated with higher mortality above the optimal temperature for a specific location. As such, the FAR method has important limitations. When there is a specific impact threshold in the system of interest, it may be informative to understand how climate change modified the likelihood that it was exceeded (Noy et al., 2024; Perkins-Kirkpatrick et al., 2022). For example, when the focus of the analysis is on a temperature threshold that results in 1,000 extra heat-related deaths, because that would strain public health resources, then a FAR analysis can be informative. However, if the focus of the analysis is on the mortality of a specific event, then a FAR analysis may be unintentionally misleading. For this reason, event attribution studies are gravitating towards approaches that assess changes in events’ intensity (e.g., temperature, precipitation) and the associated changes in the magnitude of impacts (see, e.g., Perkins-Kirkpatrick et al., 2022).

Studies have quantified climate change impacts attributable to the greenhouse gas emissions of individual entities, such as companies or countries, including for extreme weather events (Beusch et al., 2022; Lewis et al., 2019; Lott et al., 2021; Otto et al., 2017) and sea-level rise (Ekwurzel et al., 2017). Some studies quantified changes in extreme event occurrence or intensity with the emissions of individual entities excluded, for instance using reduced-complexity earth system models (Quilcaille et al., 2024). Others used simplified statistical approaches to estimate contributions proportional to the entity’s proportion of historical emissions (Stuart-Smith et al., in review).

## 5 Framework for conducting health D&A analyses: event attribution example

**Figure 1:**
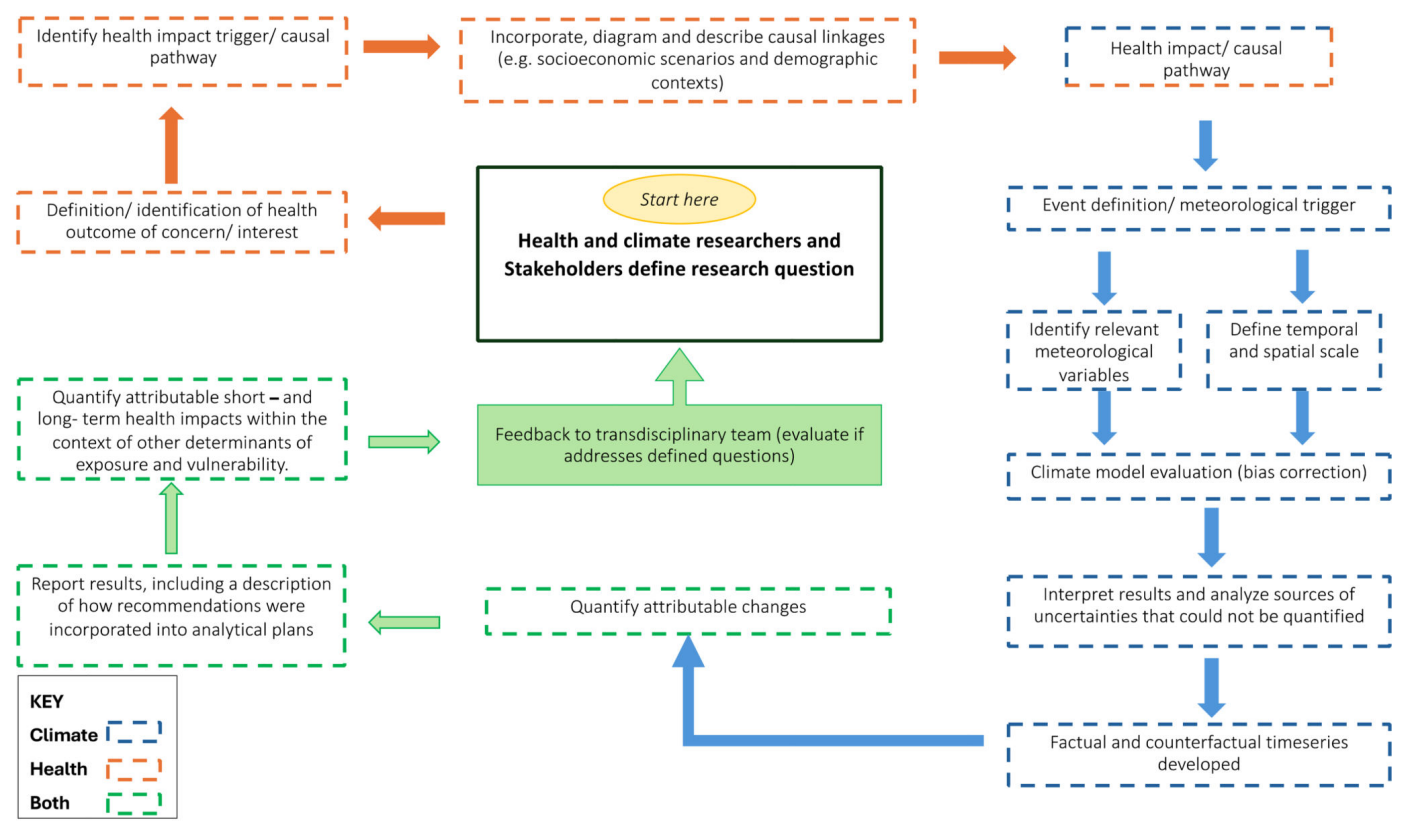
Framework for attributing impacts of extreme events to climate change. The framework is intended to be indicative of the key steps typically included in analyses rather than prescriptive. Most studies would involve similar steps and expertise.

Figure 1 outlines the framework for detecting and attributing health impacts to climate change, using extreme events as an example. Similar steps would be required for a trend D&A analytical approach, e.g. detecting and attributing health impacts to increases in temperature, changes in seasonal temperatures, or changes in the frequency, intensity, and duration of other extreme weather and climate events, and alterations in precipitation patterns (e.g., Childs et al., 2024).

In addition to the framework presented here, additional steps may be appropriate, for example considering whether other factors could have changed vulnerability or exposure, such as deforestation. Further experience in health D&A studies will refine the framework. This might include the development of standard approaches for incorporating adaptation and location- and population-specific vulnerabilities.

## 6 Guidelines

Annex 1 provides a checklist for analyses to attribute human health outcomes to climate change.

### 6.1 Work jointly across disciplines to define the research question/ intent

The research question frames all subsequent choices made. What specifically the study is set to achieve will determine who is involved, the methods used in climate and health analyses, the data required, the causal links identified, how the results are communicated, etc. (Otto 2017; van Oldenborgh et al., 2021; Perkins-Kirkpatrick et al., 2024)

### 6.2 Establish a transdisciplinary analytic team

The analytic team should include researchers with a foundational training in climate science with expertise in attribution methods, health scientists with relevant expertise, adding other disciplinary scientists or skill sets as needed to ensure the team has sufficient breadth and depth of expertise in the research disciplines and region(s) of interest. Examples include statisticians or scientists trained in applying statistical approaches elsewhere such as economists for method development, agricultural scientists for climate change-health impacts relating to food production or hydrologists for climate change-health impacts mediated via water safety and security, Indigenous Knowledge holders for climate-health impacts on Indigenous Peoples, and similar.

### 6.3 Meaningfully engage communities of practice, partners from affected communities and representative decision makers

to inform study design and enhance policy relevance and uptake. Early engagement of communities and people likely affected by the event/trend will ensure their perspectives are incorporated into the study design and the practical utility of the analyses maximized. This approach is increasingly used by some health-oriented funders in their grant-making and research priority setting processes.

### 6.4 Identify, diagram, and describe causal linkages in an evidence-based causal pathway linking exposure to weather/climate variables to the health outcome(s) of interest

The analytic team and partners should collaboratively develop a framework illustrating the drivers of the health outcome of interest, including the meteorological trigger (e.g., daily maximum temperature, or changes in the start of the spring season), and assess the evidence for detection of a climate signal for that health outcome. Causal relationships should only be assumed from exposure-response relationships with a clear theoretical basis, not just from correlational analyses between a weather variable and health outcome. Plausible mechanisms for observed results need to be described and quantified to the extent possible. The socioeconomic and demographic contexts (e.g., changes in age structure, poverty levels and the physical environment of urban and rural areas, including factors that may modify exposure-response relationships like green space or access to air conditioning) should be described, including changes over time. For example, Burden of Proof methods (Zheng et al., 2022) is one of several standardized approaches that may be useful for assessing and reporting the strength of causal evidence for a disease pathway.

A key area for improvement is the relationships between climatic stressors and observed health outcomes. A large part of the empirical literature studying climate change impacts on human health includes limited consideration of the socioeconomic and environmental factors that likely modify climate change-health pathways such as income and wealth, gender, quality of housing stock, access to electricity, and access to health care (Dasgupta and Robinson, 2024). Focusing on leverage points can help with untwining the multiple factors influencing climate change-health pathways.

### 6.5 Define the exposure event (or trend), evaluate the climate model(s) skill, and quantify attributable changes in health-relevant meteorological variables

To determine the contribution that climate change made to a health outcome, the plausible climate-related drivers of the health outcome of interest must be identified and used to define the event or trend of interest. This includes identifying the relevant meteorological variables; the geographic and temporal scales including the importance of antecedent conditions; whether changes in an event’s intensity or return period will be the focus; changes in the start and end date of the event or season of interest; and the appropriate counterfactual in which the effect of climate change is excluded. Also, where possible, studies should develop additional scenarios to address the influence of other drivers that contributed to the health outcome of interest. This includes considering natural climate variability cycles such as ENSO events and separating their influence on health outcomes from anthropogenic climate change (Haines and Lam, 2023). Health and climate data and model availability can affect the choices made.

The skill of the models should be evaluated in terms of their fidelity in capturing the appropriate, observed meteorological drivers. If the models are biased, they should be bias adjusted (e.g., ISIMIP), weighted and constrained, as appropriate. This will require evidence from relevant populations or models (e.g., physiological or epidemiological studies), with appropriate attention to generalizability and confidence intervals across the range of exposure values, to understand the relevant meteorological variables for analysis. Note that for many health outcomes, such as heat-related mortality, both events and trends are relevant, particularly when considering the distribution of potential confounders including socio-economic development patterns.

Ideally, multiple models are used to quantify attributable changes. Doing so strengthens the attribution statement. In extreme event attribution, it is now standard practice to use multiple climate models because the specific anthropogenic signal will vary across models, assessing results’ robustness to model differences. Combining model results provides a robust attribution statement. A similar approach should be the aim for health D&A models, for example by combining an epidemiological model of heat-related mortality with quantification of excess deaths. These would provide different absolute numbers of deaths that, when combined qualitatively, strengthen the attribution statement.

### 6.6 Quantify attributable short- and long-term health impacts within the context of other determinants of exposure and vulnerability

Ideally, health data are collected on the same temporal and spatial scales as exposure variables. In reality, health data are often collected on a coarser temporal and spatial scale than weather and climate data, requiring justification of necessary assumptions (Chapman et al., 2022). Typically, data are collected from national or local morbidity or mortality registries, electronic medical records or healthcare facilities, or from cohort studies. The burden of health outcomes changes over time as vulnerability, risk factors, and exposures change, and interventions to reduce the health burden are increasingly implemented. The ability of health models to capture the relationships between observed meteorological drivers and observed health impacts should be regularly assessed. Exposure-response relationships should be calculated and used at spatial scales representing the scale at which exposures are experienced.

Ideally, data are available on all factors that may affect vulnerability to both exposure (events and trends) or health outcomes. Where possible, data on population size and on characteristics that affect vulnerability to the exposure can inform meaningful counterfactual scenarios (e.g., age distribution to determine whether the proportion of the population above age 65 years increased over time, which would affect heat-related mortality). Data on other determinants of exposure can inform the analyses, such as air pollution data (or proxies) for analyses on heat-related or wildfire-related morbidity and mortality, particularly for socially and economically marginalized populations that could experience greater exposure or health impacts (e.g., changes in subpopulations in cities with less greenspace with more intense urban heat islands; political/social instability; or structure of or access to healthcare). The robustness of D&A studies would be improved by data on interventions implemented to reduce vulnerability, to assess the possible effects of adaptation and mitigation measures. The effect of mitigation measures on climatic variables should be captured by attribution analyses, but the co-benefits of mitigation actions, such as reducing air pollution morbidity and mortality, would not be captured but might affect changes in vulnerability over time. Data that consider the extent to which other environmental stressors could modify climate and health relationships, such as areas where freshwater aquifers are depleted, also could improve the robustness and relevance of D&A studies.

Analyses should document the extent to which the incidence or prevalence of the health outcome of interest changed over time and any changes in the relationships between exposures and outcomes among vulnerable and marginalized populations. Understanding the sensitivity of exposure-response relationships to changing vulnerability and exposure may inform appropriate adaptation approaches and indicate the consequences of a chosen adaptation approach. In instances in which exposure-outcome relationships are unavailable or cannot justifiably be applied to the exposure range being modeled, relationships can be estimated using standard methods (e.g. Vicedo-Cabrera et al., 2019; Gibb et al., 2023) if sufficient data are available for a valid assessment.

Models of relationships between weather/climate variables and health outcome are generally either empirical (statistical) or process-based (biological or mechanistic), each with strengths and weaknesses (Ebi 2022). Empirical models develop relationships between observations of exposure and response variables, often assuming a linear relationship. Challenges include data availability, accuracy, and length of time series. Further, observations of recent disease patterns implicitly incorporate the extent of effectiveness of control programs. Validated absence data are generally not available, which means these models may not accurately reflect the underlying relationship between an exposure and response. Mechanistic models use equations to describe the dynamics of disease etiology; challenges include poorly understood parameter values and the possibility of unknown processes. Describing the criteria to select one modeling approach over another would increase the transparency of the D&A analyses.

### 6.6 Report the results, including a description of how the above recommendations were incorporated into the analytical plan

A synthesis document or peer-reviewed publication should describe the framework used to inform the analyses; the health, climate, and other data collected; efforts to align the data on the same spatial and temporal scales; consideration of other drivers of the health outcomes; and any counterfactual scenarios used. The analytic approaches should be described and justified. General study limitations and sources of uncertainty that could not be addressed should be described.

## Discussion

Health D&A analyses can provide robust evidence to move from statements about current associations between weather variables and health outcomes to statements documenting the magnitude and pattern of current impacts of climate change on health and wellbeing. This moves the policy relevance of climate change within the health sector from a future consideration into the current mandate of ministries and departments of health. Further, D&A analyses provide a robust foundation for negotiations related to the Global Goal on Adaptation and to Loss and Damage, and for judicial adjudication in climate lawsuits.

Despite the opportunities, limited climate change and health attribution research has been conducted to date, with studies unequally spatially distributed (Carlson et al., 2024). The full range of methods from climate and health sciences have yet to be exploited. In addition, few regions and health outcomes have been analyzed in health D&A studies.

There is substantial methodological diversity and often insufficient characterization of the strength of causal evidence in pathways used for analysis. Explicit description of causal pathways and reporting of causal relationships will help address concerns related to causality, while collaborative partnerships with climate scientists would help address concerns related to situating attribution studies within continuously evolving approaches. Further, standards need to be developed for robust approaches for extrapolating data or relationships from one location to another, such as comparable climatic, demographic and socioeconomic structures, and health profiles. These standards would help scale up current analyses to provide a more comprehensive assessment of the impacts of climate change on health.

Limited consultation with affected communities and people and with decisionmakers reduces application and relevance of D&A studies. Creating an expectation of, and providing support for, such consultation by funders and incorporating this consultation into timelines for proposals and analyses could begin to address this concern. Important interactions between the frequency of extreme events, recovery times, depletion of resources for recovery, and changing thresholds for damage, such as compounding vulnerabilities from repeated extreme events, may be fruitful areas for exploration to inform decision-making (e.g., Young and Hsiang 2024).

Generating insight into the impacts of climate change on health despite data limitations remains a critical challenge that must balance retaining robustness with ensuring that large knowledge gaps are not left in understudied regions (e.g., Vicedo-Cabrera et al., 2021). Limited weather/climate and health data pervades health attribution analyses in many regions that are presumed to be highly affected by climate change (Otto et al. 2020; King et al. 2023). Stable, long-term funding to ensure the continuous collation of high-quality weather/climate and health data must therefore be viewed as a necessity to improve understanding of climate-related health risks. Until then, an opportunity for further D&A analyses is developing approaches that combine qualitative and quantitative data. In some settings, fully quantitative analyses are possible; in others, adequate and appropriate weather/climate and/or health data may be missing, permitting analyses on the climate driver alone, or on the health outcomes but not the contribution of climate change to their climate-related drivers (e.g., drought). Mixed methods approaches are being used to collect Indigenous and traditional knowledges and bridging them with Euro-western approaches to data analyses, which can be valuable (Redvers et al., 2023).

Guidance is mainly of value if it is widely implemented, therefore research funders could consider following or advancing the proposed framework and guidance as a condition of funding (or in exceptional cases a strong justification for not following the guidance). Incorporation of guidance related to D&A studies and other approaches to building evidence related to climate and health in hubs such as the Equator Network could increase uptake. Journal editors, reviewers, and judges (in climate lawsuits) can also reinforce good practice by demanding high standards of evidence generation. The proposed guidance also can support good practice in doctoral training programs and short courses in research methods.

A growing understanding of the extent to which climate change is affecting health will become increasingly central to real-world decisions on investments into and prioritization of climate and health policies and programs. For example, during the 2021 Pacific heat dome in Canada and the United States, over the course of one week, emergency departments were overwhelmed, with more than 440 excess deaths in the state of Washington (Climate Impacts Group 2023) despite accurate forecasts with lead times of 10-20 days. This event that broke all-time maximum temperature records was virtually impossible without climate change (Philip et al. 2021). The resulting impacts led to a multi-agency extreme heat response plan being developed and deployed, including technical support for healthcare systems, cooling centers, and outreach to at-risk populations (Public Health, Seattle and King County, 2024). In another example, climate change is increasing extreme flooding in Africa, which interacts with conflict, poverty, and waste management to impact vulnerable communities, underscoring the need for strengthening transboundary early warning systems through data sharing and collaboration (Pinto et al., 2024).

Open science practices would support building a larger evidence base of health D&A analyses. For example, the Coupled Model Intercomparison Project (CMIP) was, from its start, committed to making climate model simulations publicly available (e.g. through the Earth System Grid Federation, ESGF). Likewise, the ISIMIP project is producing a portfolio of climate and socioeconomic data publicly available, enabling D&A studies across a range of impact sectors (Mengel et al., 2021). Climate and impact models are increasingly open source. Without such commitments to open science, scientific progress would be much slower, in terms of model evaluation, model improvement, and attribution applications. Moreover, open science facilitates reproducibility, a key component of credible impact attribution studies, which are increasingly used in high-stake applications such as global media coverage, international climate negotiations, policy development, and climate lawsuits. For example, the Working Group I contribution to the IPCC 6^th^ Assessment Report used FAIR (Findable, Accessible, Interoperable, Reusable) data principles to facilitate open science by ensuring that the data and code used are findable and accessible and can be reused for reproducibility and for further developments using interoperable (Iturbide et al., 2022).

Fostering open science in health impact attribution studies is complicated because of national and sub-national data privacy regulations that determine who can gain access to what personal health data at what scale. These regulations are designed to protect individual privacy and have become increasingly stringent over time. The goal of these regulations is to ensure that it is not possible to identify an individual from anonymized data because of a rare condition or characteristic. Transdisciplinary research teams will need to develop strategies for merging health and weather/climate data taking these restrictions into account. Open access health data are generally annual across large geographic regions, which is generally not specific enough for D&A analyses. However, open science can be promoted by publishing articles fully open access with open-source code by researchers from high- and middle-high income countries, noting that this further disadvantages researchers from low- and middle-low income countries. This critical bias needs to be addressed explicitly and quickly to ensure knowledge and insights gained from all sources are accessible by all, to strengthen resilience to further climatic change.

Rapid advances in D&A methods in climate and impact sectors means that guidance on conducting attribution analyses should be regularly updated by incorporating insights gained through experience, changes in data availability, modeling developments, and other advances. This would future proof the guidance and promote uptake and increase its utility. Periodic and perennial systematic reviews of attribution studies are needed to assess the changing magnitude and pattern of the health effects of climate change.

## Supplementary Material

Annex

## Figures and Tables

**Table 1:** Climate model data that can facilitate D&A analyses^1^

Dataset name	Institutional Source	Weblink / reference
CMIP6 (historical, pre-industrial control simulations)	World Climate Research Programme	https://wcrp-cmip.org/ Eyring et al., 2016
DAMIP (natural-only historical simulations [hist-nat], well-mixed greenhouse-gas-only historical simulations [hist-GHG], anthropogenic-aerosol-only historical simulations [hist-aer])	Detection and Attribution Model Intercomparison Project	https://damip.lbl.gov Gillett et al., 2016
ATTRICI	Inter-Sectoral Impact Model Intercomparison Project	https://www.isimip.org Mengel et al., 2021
HiResMIP2 (see also HiResMIP1)	High Resolution Model Intercomparison Project phase 2	https://highresmip.org/ Roberts et al., 2024
Coordinated Regional Climate Downscaling Experiment (CORDEX; and other coordinated regional modelling experiments)	WCRP CORDEX	https://cordex.org Giorgi et al., 2009

1 Note that this table is neither intended to be complete or representative of the model datasets used in climate change attribution studies. DAMIP is most commonly used across the literature. Region-specific semi-operational attribution tools are also being developed by national meteorological services (e.g., UK, Japan, France, Canada, Australia, New Zealand).
