# Supplementary material for: The attribution of human health outcomes to climate change: a transdisciplinary guidance document
Source: Clim Change. Author manuscript; Available in PMC 2025 Sep 17. (PMC7618133; doi:10.1007/s10584-025-03976-7)
Supplement: Annex [file EMS208690-supplement-Annex.pdf]

Annex 1: Checklist for attribution of human health outcomes to climate change

Define the research question

- Describe the event/trend of interest, including its geographic and temporal scale.
- State the study aims and objectives.
- Specify the target audience(s) including primary decisionmakers of interest.

Establish a transdisciplinary analytic team

- Include researchers with a foundational training in climate science with expertise in attribution methods, health scientists with relevant expertise at a minimum, adding other disciplinary scientists or skill sets as needed to ensure the team has sufficient breadth and depth of expertise. Provide a rationale for which disciplines should be included
- Include representatives from communities of practice, partners from affected communities, and decisionmakers, including experts on the region(s) of interest as relevant.
- Outline the causal links between the event/trend and health outcomes, including
    - Major determinants of susceptibility/vulnerability to climate change (e.g., population aging, socioeconomic status)
    - Effect modifying factors that may influence the climate-health relationship, including other environmental changes e.g. urbanization and other land use change.
- List and describe the data required and justify the analytic methods to be used.
- Establish a communication plan.
- Publishing the study protocol in advance of the analyses will improve research standards by promoting transparency, reduce publication bias, and enhance the reproducibility of study design and analysis.

Establish a plan to meaningfully engage with communities of practice, partners from affected communities and representative decision makers as the analyses are conducted

- Describe strategies to facilitate appropriate stakeholder engagement and communication with the target audience. Early engagement of communities and people likely affected with ensure their perspectives are incorporated.

Identify, diagram, and describe causal linkages in an evidence-based causal pathway linking exposure to weather/climate variables to the health outcome(s) of interest

- Develop a framework illustrating the drivers of the health outcome of interest, including the meteorological trigger where relevant and assess the evidence for detection of a climate signal for that health outcome.
    - Use causal relationships based on rigorous research describing exposure-response relationships at appropriate spatial and temporal scales, accounting for lagged effects where appropriate
- Describe and quantify plausible mechanisms for observed results, to the extent possible.
- Describe the socioeconomic and demographic contexts (e.g., changes in age structure, poverty levels) and changes in the physical environment of urban and rural areas, including changes over time that could affect the health outcomes of interest.

Define the exposure event (or trend), evaluate the climate model(s) skill, and quantify attributable changes in health-relevant meteorological variables

- Determine the contribution that climate change made to the health outcome. This includes identifying the relevant meteorological variables; the geographic and temporal scales including the importance of antecedent conditions; whether changes in an event's intensity or return period will be the focus; (changes in) the start and end date of the event or season of interest; and the appropriate counterfactual in which the effect of climate change is excluded
- Justify why the attribution approach used (for instance, probabilistic or storyline methods; presenting results as changes in intensity and/or probability) is appropriate for the impact pathway being studied.
- Where possible, develop additional scenarios to address the influence of other drivers that contributed to the health outcome of interest., such as natural climate variability exemplified by El Niño events.
- Evaluate the skill of the models in terms of their fidelity in capturing the appropriate, observed meteorological drivers. If model biases are identified, they should be bias corrected, weighted and constrained, as appropriate.
- Use multiple climate models to quantify attributable changes where possible, to strengthen the attribution statement and reduce results' sensitivity to a single model.
- Describe and analyze sources of uncertainty that could not be addressed quantitatively.

Quantify attributable short- and long-term health impacts within the context of other determinants of exposure and vulnerability

- Ideally, health data can be collected on the same temporal and spatial scales as exposure variables, although that is uncommon in practice.
    - For health data at coarser temporal and spatial scales than weather and climate data, justify necessary assumptions.
- Describe and justify exposure-response functions used.
    - Where possible, describe how these functions vary among vulnerable and marginalized populations.
- Define and justify the health metrics used, such as mortality, disability-adjusted life years lost, and years of life lost.
- Define and justify the data sources used.
- Assess the ability of the health model(s) to capture the relationships between observed meteorological drivers and observed health impacts.
- Where possible, use data on population size and on characteristics that affect vulnerability to the exposure to inform meaningful counterfactual scenarios (e.g., age distribution to determine whether the proportion of the population above age 65 years increased over time, which would affect heat-related mortality).
- Include data on other determinants of relevant exposures to inform the analyses, such as air pollution data (or proxies) for analyses on heat-related or wildfire-related morbidity and mortality.
    - Take into account the possible effects of adaptation and mitigation measures on exposure-response relationships.
- Document the extent to which the incidence or prevalence of the health outcome of interest changed over time and any other changes in the relationships between exposures and outcomes among vulnerable and marginalized populations. In instances in which exposure-outcome relationships are unavailable because of data limitations or cannot justifiably be applied to the exposure range being modeled and so need to be extrapolated, the assumptions used should be clarified and sensitivity testing employed to assess the effects of varying assumptions.

- Describe and analyze sources of uncertainty that could not be addressed quantitatively.

Report the results, including a description of how the recommendations were incorporated into the analytical plan

- A synthesis document or peer-reviewed publication should describe the framework used to inform the analyses; the health, climate, and other data collected and their sources; approaches to align the data on the same spatial and temporal scales; consideration of other drivers of the health outcomes; and any counterfactual scenarios used. The analytic approaches should be described and justified. General study limitations and sources of uncertainty that could not be addressed quantitatively should be described.

Data and Code Transparency

- Where possible, openly and publicly share data and code to facilitate open science.
    - Legal or ethical limitations to data sharing should be explicitly stated.
